# Refractive Index Mapping below the Diffraction Limit via Single Molecule Localization Microscopy

**DOI:** 10.1021/acsnano.5c17647

**Published:** 2025-12-26

**Authors:** Simon Jaritz, Lukas Velas, Anna Gaugutz, Manuel Rufin, Philipp J. Thurner, Orestis G. Andriotis, Julian G. Maloberti, Simon Moser, Alexander Jesacher, Gerhard J. Schütz

**Affiliations:** † Institute of Applied Physics, 27259 TU Wien, 1060 Vienna, Austria; ‡ Institute of Lightweight Design and Structural Biomechanics, TU Wien, 1060 Vienna, Austria; § Institute of Biomedical Physics, 27280 Medical University of Innsbruck, Müllerstraße 44, 6020 Innsbruck, Austria

**Keywords:** 3-dimensional single molecule localization microscopy, dSTORM, defocused imaging, multimodal imaging, refractive index, collagen fibrils, atomic force microscopy

## Abstract

Single molecule localization microscopy (SMLM) is a powerful method to image biological samples in three dimensions, below the diffraction limit of light microscopy. Beyond the position of the emitter, the shape of the single-molecule point spread function provides additional information, for example, about the refractive properties of the sample between the emitter and the glass coverslip. Here, we show that the combination of SMLM with atomic force microscopy (AFM) allows mapping of the refractive index of a biological sample at subdiffraction resolution and at a precision only limited by measurement errors of SMLM and AFM. We showcase the method by the determination of the refractive index of isolated single collagen fibrils. Variabilities both in refractive index and the swelling behavior of single fibrils upon drying and rehydration exposed deviations from the ensemble behavior, demonstrating differential hydration of single collagen fibrils. Mapping the refractive index along single collagen fibrils revealed substantial fluctuations at characteristic length scales below 500 nm, which indicates the structural heterogeneity of collagen fibrils at the length scale of single collagen molecules.

## Graphical abstract

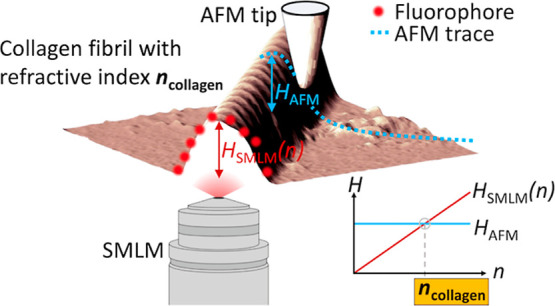

Single-molecule localization microscopy has boosted our information on the spatial organization of biological samples, especially cells.^1^ The overall idea is to employ rare stochastic appearances of single molecule signals in order to separate them from those of neighboring molecules, which allows circumvention of the diffraction limit of light microscopy. Appearances can be due to the stochastic blinking of dye molecules in STORM or PALM or the stochastic binding of fluorescently labeled ligands in PAINT. The obtained signals then allow for determining the 2D center of mass as a proxy for the 2D position with a precision that is mainly limited by the signal-to-noise ratio. At high signal quality, localization precisions below 1 nm have been reported.^2^

Also, the shape of the single molecule point spread function (psf) contains valuable information. For example, its width allows for extracting a dye’s position along the optical axis.^3^ This can further be improved by introducing artificial astigmatism^4^ or other phase-shaping methods.^5^

We have recently introduced defocused imaging in combination with supercritical angle detection, which allows for achieving impressive localization precision down to 10 nm in case emitters are located close to surfaces of high refractive index.^6^ In addition, the psf shape also varies with dipole angle, which has been used to determine azimuth and inclination angle precisely.^7^

The above-mentioned research problems are all well-conditioned in the sense that the input argument’s variation has a distinct effect on the psf shape. In contrast, there are also ill-conditioned problems where the variations of different parameters have nearly identical effects on the psf shape. One example is the refractive index *n* of the sample, which affects the psf similarly to the distance from the focal plane. Therefore, if *n* was not considered correctly, the calculated axial distances will be distorted compared to the true distances.^8^ Conversely, psf analysis could also be used to determine the refractive index of the sample if the axial distance was known.

We reasoned that a bimodal imaging approach, in which SMLM is combined with a second imaging modality to record the axial position of the dyes, would transform the ill-conditioned problem into a well-conditioned problem for refractive index mapping at a resolution below the diffraction limit of light microscopy. For proof-of-principle, we used here a combination of SMLM with atomic force microscopy (AFM) to determine the refractive index of collagen fibrils mounted onto glass coverslips. For the achieved signal-to-noise ratio, the method allows for determining the refractive index to a precision of σ_*n*_ < 3 × 10^–3^. We applied the method to compare the refractive index of differently swollen collagen fibrils: with increasing water content, we observed the refractive index of single collagen fibrils to decrease toward the refractive index of water.

## Results

The concept of super-resolved refractive index mapping is sketched in Figure 1a. A fluorescently labeled sample with refractive index *n*_s_ is mounted on a glass coverslip with refractive index *n*_g_ and imaged both with AFM and SMLM. AFM provides the ground truth information about the distance of the fluorophores from the coverslip surface, *H*_AFM_. Next, SMLM is performed on the very same sample. Figure 1b shows two hypothetical psf profiles calculated for a distance of *H* = 100 nm from the coverslip surface, which differ only in the refractive index of the underlying material: here, we compared *n*_s_ = 1.35 with *n*_s_ = 1.48. The difference image in Figure 1c shows a broadened psf for the higher refractive index. Without independent information on the sample’s refractive index, one would hence wrongly estimate the fluorophore’s distance from the coverslip surface, *H*_SMLM_(*n*_s_): an erroneous assumption of a refractive index smaller than the correct value would yield an underestimation of the molecule’s distance and vice versa. *n*_s_ can therefore be derived by variation in the molecular image model to achieve an optimal fit to the observations. This is possible because the molecules’ distance to the coverslip is known from independent AFM measurements, *H*_AFM_.

**Figure 1:**
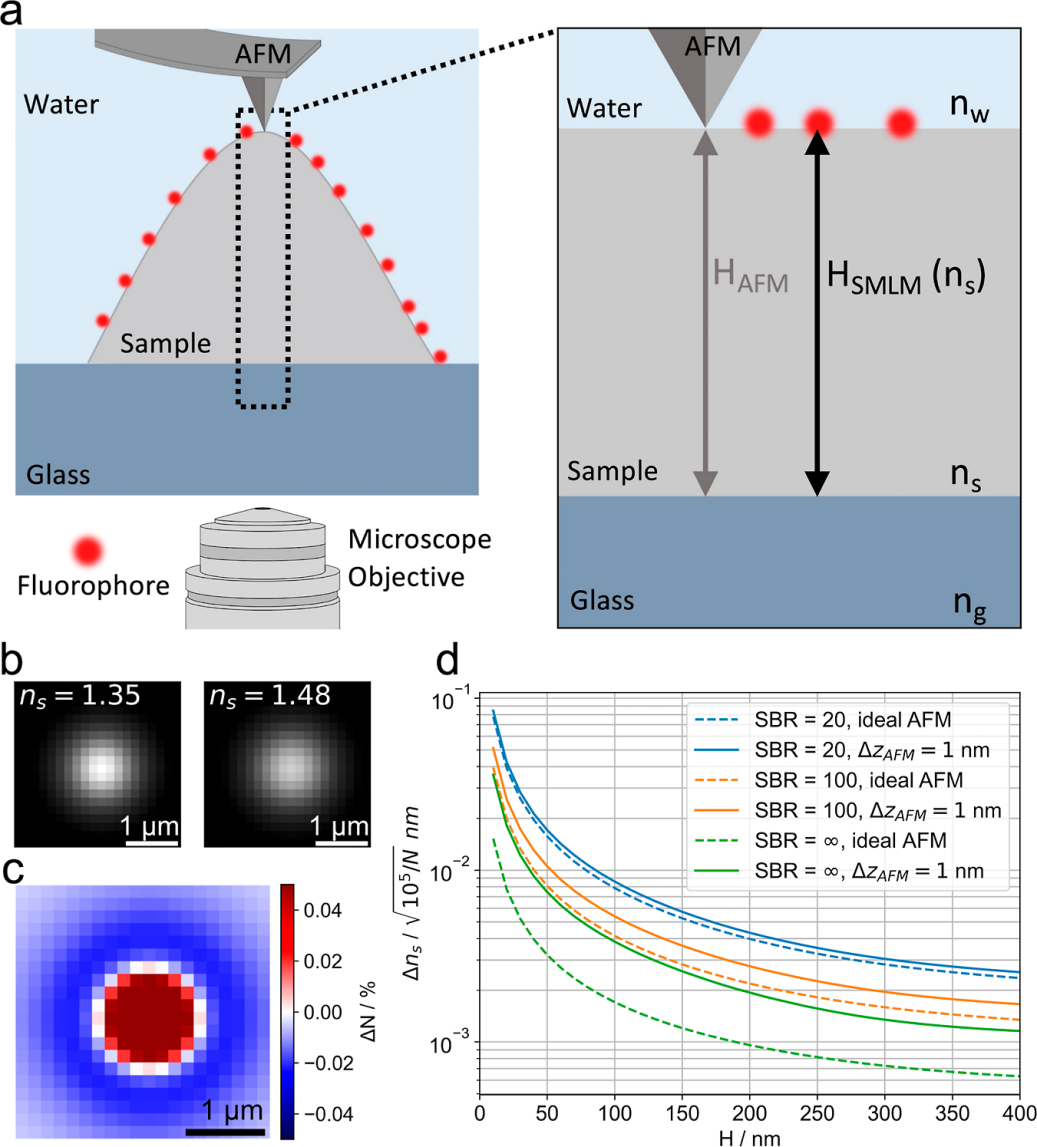
(a) Principle of the refractive index determination method. A biological sample of refractive index *n*_s_ is surface labeled with dye molecules (red dots). Its apparent height is measured by both SMLM and AFM, yielding *H*_SMLM_ and *H*_AFM_, respectively. Due to refraction within the sample, the determined value for *H*_SMLM_ depends on the assumed refractive index. Comparison with the ground truth value *H*_AFM_ hence allows calculating the sample refractive index. (b) psf for a freely rotating dye molecule located 100 nm above the glass surface and defocused by 500 nm. The left and right images were calculated for a refractive index *n*_s_ = 1.35 and *n*_s_ = 1.48, respectively. The difference image (c) shows the broadening of the psf with increasing *n*_s_ = 1.35. For the figure we assumed an ambient refractive index of water (*n*_w_ = 1.33) and a wavelength of λ = 670 nm. (d) Cramér–Rao lower bound, Δ*n*_s_, for refractive index estimates as a function of collagen thicknesses *H*. Different colors represent different signal-to-background ratios (SBR), as stated in the legend. Dashed lines assume an ideal, error-free AFM. Solid lines assume an AFM measurement uncertainty of 1 nm.

First, we were interested in the principal precision for determination of refractive indices using our approach. For this, we performed a numerical analysis based on the Cramér–Rao Lower Bound (CRLB). Figure 1d visualizes the CRLB for determination of the refractive index, Δ*n*_s_, for a sample thickness *H* between 10 and 400 nm and different signal-to-background ratios (SBR). SBR is defined here as the ratio of the signal photons to the mean number of background photons in a single pixel, assuming Poissonian noise. As the precision scales inversely with the square root of the detected signal, CRLB can be adapted to specific measurements by dividing the values by $\sqrt{\frac{N}{10^{5}}}$ , where *N* is the number of collected signal photons in the specific measurement. The plot hence directly reports on Δ*n*_s_ for *N* = 10^5^ photons, a value that can be easily obtained with SMLM. As expected, the data indicates that arbitrary precision Δ*n*_s_ can be reached if only the number of detected photons and SBR are sufficiently large. Of note, Δ*n*_s_ decreases with increasing sample height *H*, since Δ*n*_s_ scales approximately with the relative precision for determining *H*, $\frac{{\Delta H}_{SMLM}}{H}$ (Figure S1).

For an experimental demonstration, we determined the refractive index of isolated single collagen fibrils derived from mouse tail tendons. Collagen fibrils were immobilized at low density on glass coverslips and fluorescently labeled via AF647-conjugated primary antibody against collagen type I (the most abundant collagen type found in tendon collagen fibrils), giving rather homogeneous surface staining of the fibrils. SMLM imaging was performed at a defocus of appr. 500 nm, which is appropriate for optimal localization precision.^9^ From repeated observations of the same fluorescence molecules, we calculated an axial single-molecule localization precision of approximately 5 nm (standard error of the mean of all merged localizations). An overlap of all axial single-molecule observations of an exemplary collagen fibril is shown in Figure S2a. For estimation of the fluorophores’ axial positions, we varied the assumed refractive indices of the collagen fibril, which strongly affected the height of the cross-sectional profile. We quantified the fibril height from smoothened data at the crest of the fibril, yielding an apparent height *H*_SMLM_(*n*_s_) (see Methods and Experimental for details), which depends on the assumption of the refractive index *n*_s_. Next, we recorded the very same collagen fibril regions using AFM (Figure S2b) and determined the ground truth height *H*_AFM_. Comparison of *H*_SMLM_(*n*_s_) with *H*_AFM_ allowed for calculating the refractive index of the studied collagen fibril (Figure S2c). For this particular collagen fibril, we obtained *n*_collagen_ = 1.437 ± 0.003, which is in good agreement with refractive indices reported for hydrated collagen films.^10^ Here, we determined *H*_SMLM_ from a sliding window containing 40 localizations, yielding in total *N* = 123,648 photons contributing to the determined height of the cross-sectional profile. With the obtained SBR = 110 and the height of the fibril *H*_AFM_ = 138 nm, we calculate the theoretical CRLB for the refractive index of Δ*n*_s_ = 0.0034, in good agreement with the experimentally obtained value σ_*n*_ = 0.0029.

Collagen fibrils swell upon hydration, resulting in a cross-sectional area increase by up to a factor of 4 compared to the air-dried state.^11^ When submerged in aqueous solution, a substantial amount of free (unbound) water fills the intermolecular space within the collagen fibril. Because of the high dielectric constant of water, this reduces the free energy between collagen molecules, which manifests as an increase in the intermolecular distance^12^ and therefore swelling. Consequentially, the degree of swelling also influences the refractive index of collagen, with a convergence toward the refractive index of water for highly swollen collagen.^13^ However, current data are only available for average refractive indices obtained from either collagen-rich tissues like the cornea^14^ or from collagen fibers^13b^ and collagen films^10^ but not at the level of single collagen fibrils.

A number of factors influence the degree of hydration of single collagen fibrils, including cross-linking of neighboring collagen molecules.^15^ Indeed, when comparing the same collagen fibrils recorded in dried and wet conditions, we observed an increase in the cross-sectional area varying between 150% and 350%. Accordingly, also the refractive indices obtained for different fibrils varied strongly, ranging from *n*_collagen_ = 1.38 up to *n*_collagen_ = 1.48 (Figure 2a). Data from different regions of the same collagen fibril are shown in the same color. When we plotted the calculated refractive index for each collagen fibril versus its area change upon hydration, Δ*A*, we observed a strong negative correlation: in general, the higher the swelling, the lower the refractive index under hydrated conditions. This is in agreement with a model in which water incorporates into the fibrillar structure upon swelling, resulting in an increase of the intermolecular distance between neighboring collagen molecules;^11a,12^ accordingly, *n*_collagen_ approaches the refractive index of water for strongly hydrated samples.^13^ Still, we observed a substantial spread of the data, which exceeded the precision of our method, indicating that the degree of swelling is not the only determinant of collagen refractive index. Even more so, in Figure 2a, we indicated in dashed lines the predicted decrease of *n*_collagen_ with increased swelling. For this, we assumed *n*_collagen_ to represent the area-weighted average of the refractive index of dry collagen, *n*_dry_, and water, *n*_water_ = 1.33

1 $$n_{collagen}=\frac{n_{dry}+\Delta A\cdot n_{water}}{1+\Delta A}$$

**Figure 2:**
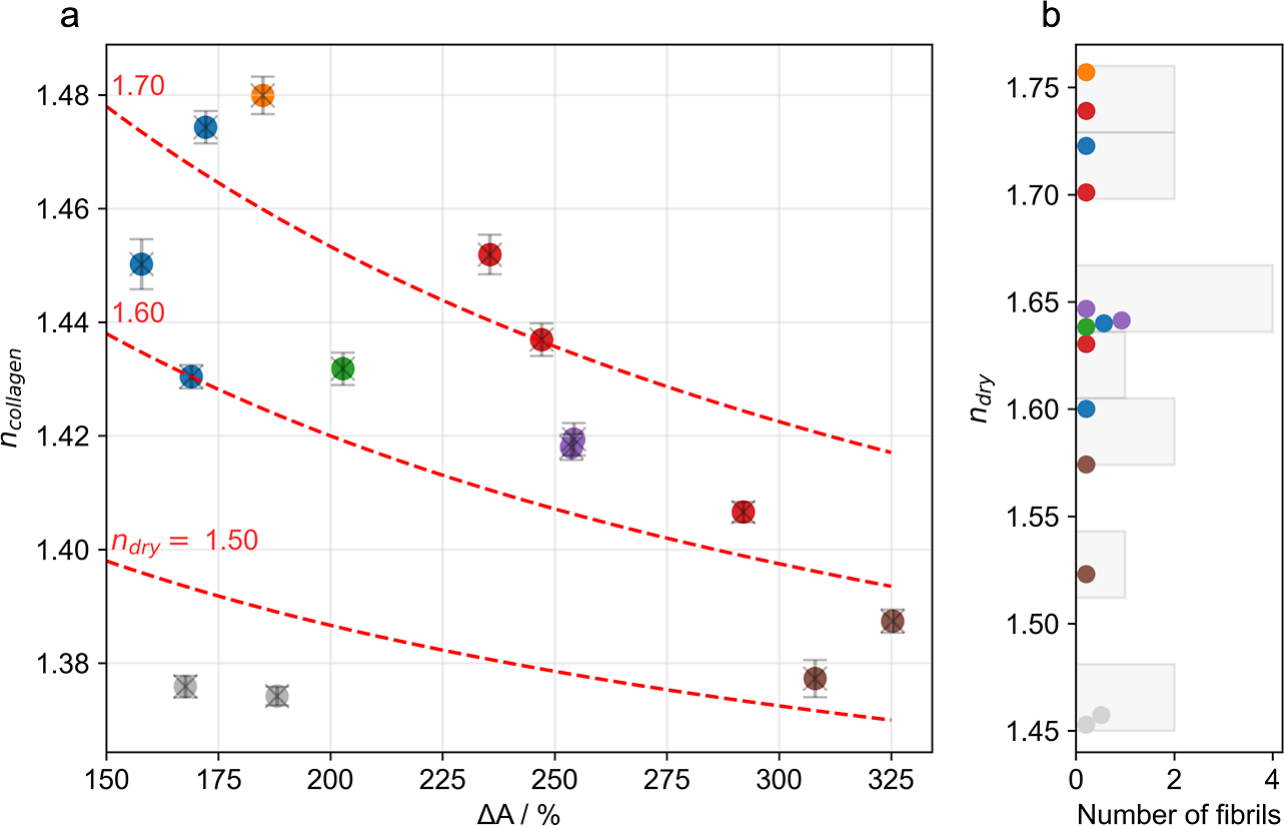
(a) Refractive index of single hydrated collagen fibrils, *n*_collagen_, as a function of the swelling upon rehydration, Δ*A*. Same colors indicate different regions analyzed on the same fibrils. Red dashed lines show the expected dependence of *n*_collagen_ on Δ*A* using eq 1, assuming different refractive indices for collagen in the dry state, *n*_dry_ (red numbers). (b) Histogram of the calculated refractive index for dry collagen, *n*_dry_, using eq 1 for the data shown on the right panel. Data are from 3 independent experiments.

Apparently, this simple model fails to describe the data. We believe the deviation to be a consequence of the violated ergodicity of the collagen sample, evident also from previous work^11d^ different collagen fibrils—albeit from the same source—can be expected to vary in the degree of dehydration that is achieved by drying the sample, potentially due to differences in the amounts of cross-links between neighboring collagen molecules. As a general trend, smaller amounts of cross-links will (i) lead to larger swelling upon rehydration,^11d^ resulting in larger values of Δ*A*, and (ii) higher degrees of residual wetting of collagen molecules in the dry state, resulting in smaller values of *n*_dry_. In Figure 2b, we showed the estimated spread of *n*_dry_, yielding 1.45 ≲ *n*_dry_ ≲ 1.75.

Finally, we investigated how the refractive index varied along a single collagen fibril. Indications for refractive index variations were already visible in Figure 2a, apparently, the refractive indices of single collagen fibrils varied more than the respective error bars. In Figure 3 we show an exemplary collagen fibril (overview, see Figure 3a) and the mapped refractive index along the central axis within a sliding window of 100 single molecule signals; the width of the sliding window corresponds to a lateral resolution of the refractive index map of 250 nm (Figure 3b). The colors and positions of the dots indicate the determined refractive index and maximal positions of the corresponding height profiles, respectively. In order to facilitate the display of the refractive index map, we stretched the image perpendicular to the fibril axis approximately 10-fold; thereby, it is possible to see, at the same time, the variation of *n* along and perpendicular to the fibril.

**Figure 3:**
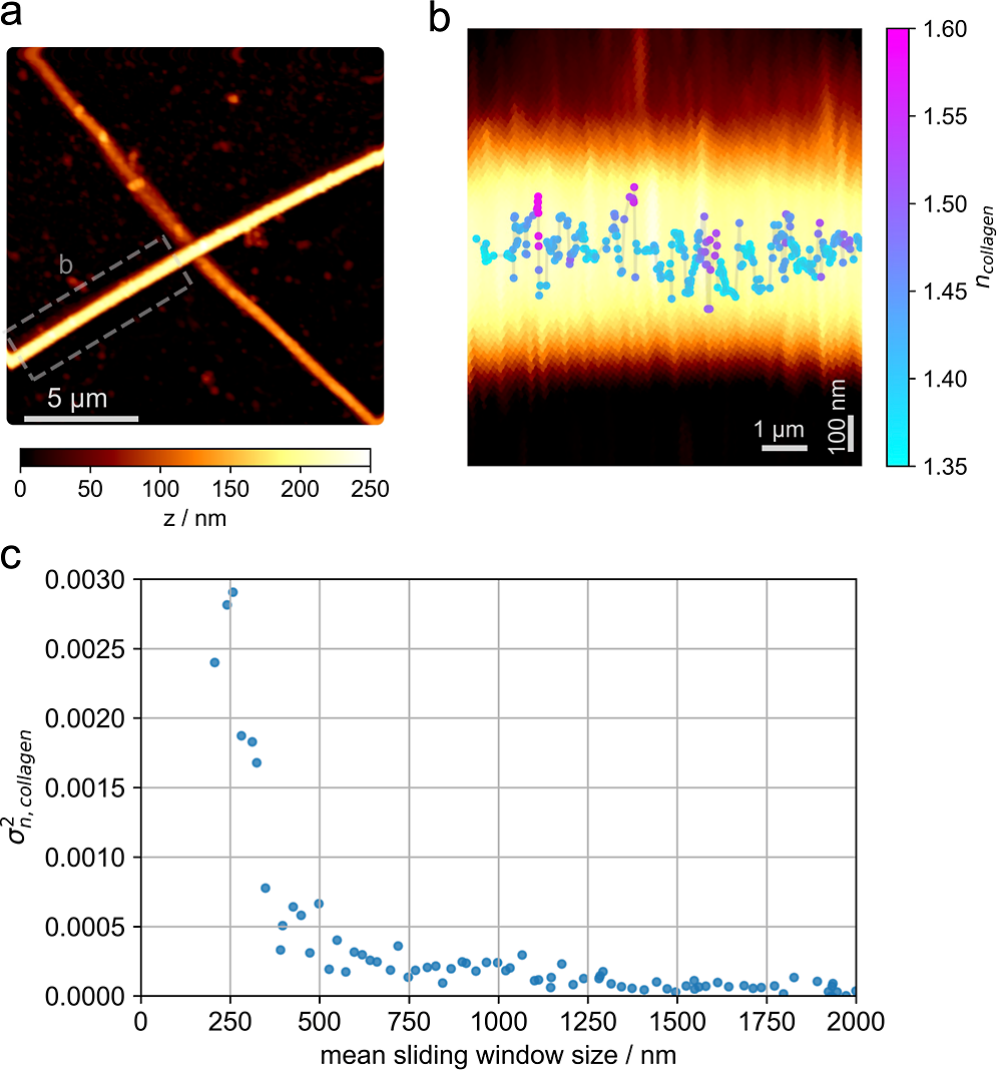
(a) Exemplary AFM image of two collagen fibrils in hydrated conditions. (b) Overlay of the AFM image from the indicated region in a, and the refractive index *n*_collagen_ along the central fibril axis (top view). Each data point was calculated from a cross-sectional profile within a sliding window containing 100 localizations, with a 90% overlap between windows; the mean sliding window size was 250 nm. In each window, *H*_SMLM_ was determined, as described in the Methods and Experimental section. Each dot was plotted at the calculated mean position of all localizations within each window. Note the approximately 10-fold stretching of the image perpendicular to the fibril axis. (c) Variance of the refractive index along the fibril shown in panel b for different sizes of the sliding window. Experimentally determined variances, σ_*n*_^2^, were corrected for experimental errors σ_*n*,exp_^2^ according to σ_*n*,collagen_^2^ = σ_*n*_^2^ – σ_*n*,exp_^2^. σ_*n*,exp_^2^ was calculated separately for each sliding window (see Methods and Experimental section).

Apparently, the refractive index varies substantially in a range between 1.35 and 1.60, indicating differently hydrated regions along the collagen fibril. To study the characteristic length scale of the refractive index fluctuations, we plotted the variance, σ_*n*_^2^, for different sizes of the analysis window. Importantly, both experimental noise, σ_*n*,exp_^2^, and refractive index fluctuations of the collagen fibril itself, σ_*n*,collagen_^2^, may contribute to the obtained fluctuations. Since the two noise contributions are uncorrelated; however, they can be disentangled, and the total variance is given by σ_*n*_^2^ = σ_*n*,collagen_^2^ + σ_*n*,exp_^2^. Furthermore, the experimental noise σ_*n*,exp_^2^ for each sliding window size can be calculated (see Methods and Experimental, Refractive Index Error Estimation), which allows for determining σ_*n*,collagen_^2^ = σ_*n*_^2^ – σ_*n*,exp_^2^ (Figure 3c). For convenience, we show on the *x*-axis instead of the window size the corresponding mean distance along the collagen fibril. Interestingly, the variance shows a steep transition from large values at distances <500 nm to rather small values at larger distances. In view of the length of single collagen molecules of 300 nm, our data hence indicates substantial variability in collagen hydration at the length scale of single collagen molecules within collagen fibrils. Similar results are shown for a second fibril in Figure S3.

## Discussion

In this paper, we describe a method to extract information on refractive properties of the sample medium from the psf shape. For this, we utilized the possibility to convert an a priori ill-conditioned into a well-conditioned problem, employing additional complementary information. Here, we showcase the method by calculating the average refractive index of the sample at high precision and by mapping the refractive index at lower precision; in our example, the additional information comes from AFM height recordings of the sample. In the following, we describe (i) the limitations of our method and (ii) its pros and cons compared to other methods for refractive index determination. Finally, in point iii, we discuss our results in the context of collagen properties.

(i) )­Limitations in the precision for determining n. In principle, the main limitation of the method is set by the accuracy with which height can be measured. In our example, we demonstrate the method with STORM recordings, yielding an axial localization precision of ∼5 nm. An alternative method would be DNA-PAINT, which can achieve even higher localization precision due to basically unrestricted amounts of localizations that can be merged to infer the position of a fluorescent label.^2^ Next, also the type of labeling needs to be considered. Here, we employed primary antibodies directly conjugated to dye molecules. We referenced the positions of the antibodies at the apex of single collagen fibrils against the positions of antibodies unspecifically bound to the glass coverslip. Reducing the label size would allow for further improving the precision of the method. In addition, one needs to rule out other effects on the psf shape. For example, it has to be ensured that the dye molecules rotate freely, as azimuth and inclination angles also affect the psf shape.^7^ This becomes even more pronounced when analyzing supercritical angle fluorescence.^16^ Here, we used freely rotating dyes conjugated to antibodies in order to avoid orientation biases. Moreover, residual aberrations of the imaging system will affect the psf shape. We corrected for such aberrations in our study using psf recordings of fluorescent beads. Eventually, one may analyze much smaller samples than those of the chosen collagen fibrils. However, height calculations both from AFM and SMLM show precisions that are almost independent of the actual height of the sample. Therefore, the thinner the sample gets, the higher the relative errors will be, and the more signal photons need to be collected to obtain a given minimum precision for Δ*n*. In Figure S4, we show the required number of collected photons for the determination of the refractive index at a precision of Δ*n*_s_ = 10^–2^ as a function of sample thickness. We see that in the case of an AFM measurement with an error of 1 nm and a realistic SBR of 100 in SMLM, about 10^5^ photons must be collected to achieve the defined minimum precision for samples of 50 nm thickness. This corresponds to >10^2^ localizations at an average signal of 10^3^ photons, which is within realistic limits. The refractive index of samples with a thickness below 40 nm can be determined to an accuracy of only Δ*n*_s_ = 10^–2^, if ground truth information on the sample height is known with better precision than 1 nm. Finally, in its current implementation, the method is only sensitive to the average refractive index between the dye labels and the glass surface. Potential axial sample heterogeneities will hence be averaged out. A particular case is samples being lifted up from the glass surface by a distance *d*, so that the determined values *n*_s_ would also include refractive index contributions from water between the sample and glass slide. This case, however, can be easily accounted for by utilizing dye labels at the bottom surface of the sample, which allow for measuring *d*

(ii) Alternative methods to determine the refractive index of biological samples at high spatial resolution: The refractive index affects various optical properties, which can be experimentally assessed.^17^ It is hence not surprising that a variety of methods exist which allow for determination of the refractive indices of biological samples. A common technique is optical coherence tomography (OCT), where differences between the optical and the physical path length are determined and used for calculating *n*.^18^ While this method provides noninvasive insights into the optical properties of samples, its sensitivity and spatial resolution are insufficient to characterize samples with sizes of 100 nm or less. Also, ellipsometry is frequently used to determine the refractive index of thin samples,^19^ albeit at classical diffraction-limited resolution. Coupling to a near-field tip can in principle improve the resolution below the diffraction limit, yet the quantitative effect of the tip presence on the determined refractive index is not fully understood yet.^20^ Recently, interferometric detection of scattering (iSCAT) was applied to calculate the refractive index of subdiffraction objects, based on their scattering contrast.^21^ Similar to SMLM, iSCAT contrast depends both on *n* and on the size of the particle *d* and hence requires an independent measurement of *d*. In ref 21, single particle tracking was used to estimate *d* of the spherical objects based on the measured diffusion constant, using Stokes–Einstein theory.

(iii) Discussion on the obtained values of *n*_collagen_. Our data on hydrated single collagen fibrils are generally in good agreement with refractive indices, as obtained for various collagen samples.^10,13a,14^ For example, Wang and colleagues measured the refractive index of collagen films via OCT and obtained *n* = 1.43 and *n* = 1.53 for hydrated and dehydrated samples, respectively.^10^ A larger spread was observed for fascicles also via OCT, yielding *n* = 1.37 and *n* = 1.57 for hydrated and dehydrated samples.^13a^ However, all results reported so far were obtained on rather large collagen samples, where data correspond to an average refractive index over multiple collagen fibrils. This precluded direct correlation between refractive index and swelling behavior upon hydration. Our high-resolution imaging approach, in contrast, allowed us to correlate the single fibril refractive index with its particular swelling behavior. We found a surprising heterogeneity both in the swelling behavior and in the refractive index, potentially due to different amounts of intermolecular cross-links within single fibrils.^11d,22^ From the degree of swelling and the refractive index of water, we estimated the expected refractive index of each fibril in the dry state, yielding a spread from *n*_dry_ = 1.45 up to *n*_dry_ = 1.75. We interpret the rather large variation in refractive index as a consequence of variations in the residual hydration of collagen in the dry state, potentially resulting from variations in the amounts of intermolecular cross-links: in this view, a low refractive index would correspond to collagen fibrils with weakly cross-linked collagen molecules, showing a higher degree of residual hydration at the air-dried state; a high refractive index, in contrast, would relate to collagen fibrils with well-cross-linked collagen molecules, from which unbound water was efficiently expelled during drying. We observed this variability in refractive index even at the level of single collagen fibrils with a strong distance dependence: *n*_collagen_ varies strongly at length scales below 500 nm along the fibril axis and less at larger distances. This behavior may indicate variations in the amount of intermolecular cross-links at the level of single collagen molecules. Single collagen molecules are 300 nm in length and twisted into a triple helix. Upon translation, collagen molecules undergo several modifications at the ER, including the enzymatic hydroxylation of lysine. Variability in the degree of lysine hydroxylation between different collagen molecules can hence be expected. Importantly, the presence of hydroxylysine is a prerequisite for the formation of a class of covalent cross-links between adjacent collagen molecules during fibrillogenesis in the extracellular matrix.^23^ Together, the propensity of collagen to form intermolecular cross-links likely varies on the length scale of approximately 300 nm, which would lead to corresponding variabilities in the local hydration and, hence, refractive index. Finally, it is interesting to compare our results to refractive indices of other proteins, as obtained at different humidities. For green fluorescent proteins, e.g., the refractive index increased from 1.45 to 1.75 from the hydrated to the dry state,^24^ in good agreement with the predictions of our study.

## Conclusions

We report a method for refractive index mapping at a spatial resolution below the diffraction limit of optical microscopy that exploits the dependence of the point spread function shape on the refractive index of the sample. It requires the combination of superresolution fluorescence microscopy with a nonoptical high-resolution imaging technique, in our case atomic force microscopy, to independently record sample thickness with two different imaging modalities, where only one is affected by the sample refractive index. Knowing a sample’s refractive index is of high interest for understanding its structural organization, particularly at length scales below the diffraction limit of light microscopy. Our method allows us to obtain this parameter at unprecedented spatial resolution combined with high precision. Given the general availability of both single-molecule localization microscopy and atomic force microscopy in many imaging laboratories and facilities, the application of the method appears straightforward. Here, we applied it to study the refractive index of collagen fibrils. We observed unexpected fibril-to-fibril variabilities upon swelling, indicating variabilities in the amount of residual water contained in dry fibrils. Eventually, we mapped the collagen refractive index along the axis of single collagen fibrils, revealing fluctuations at typical distances below 500 nm. We speculate that both fibril-to-fibril variabilities and single fibril fluctuations of the refractive index can be linked to variabilities in the amount of interfibril cross-links.

## Methods and Experimental

### Materials

- PBS (DPBS, D8537-1L, Sigma-Aldrich)
- anti-collagen1­(I) antibodies, Alexa Fluor 647 conjugated (antibodies, Rabbit, polyclonal, bs-10423R-A647, Bioss)
- BSA (A3803-10G, Sigma-Aldrich)
- PFA (stock solution 16% PFA, methanol free, MP Biomedicals)
- glucose, glucose oxidase, catalase, cysteamine (Sigma-Aldrich).
- precision tweezers (fine tips, Inox08,5, Dumont)
- coverslip (24 × 60 mm, #1.5, Menzel)
- dental glue (Twinsil extra hard, Picodent)
- Fluorescent beads (FluoSpheres, Cat. Nr. F8780)
•Mouse tails (wild type, 6 months old, female; provided by Peter Pietschmann (Medical University of Vienna, Austria))

All animal procedures were conducted in accordance with institutional and national guidelines. Under the Austrian Federal Act on Experiments on Live Animals (Bundesgesetz über Versuche an lebenden Tieren—Tierversuchsgesetz 2012—TVG 2012, Bundesgesetzblatt Nr.: BGBl. I Nr. 114/2012.), sacrificing animals for the sole purpose of harvesting tissues for use in further experiments is not regarded as a regulated (the federal law) animal experiment. Thus, permission from the Ministry of Health for this activity is not required as described in §2 (Begriffsbestimmungen—“Definitions”). The link to the federal act—TVG2012 is https://www.ris.bka.gv.at/GeltendeFassung.wxe?Abfrage=Bundesnormen&FassungVom=2013-07-10&Gesetzesnummer=20008142&ShowPrintPreview=True&utm_source=chatgpt.com.

Tissue were harvested at the Medical University of Vienna at the Institute of Pathophysiology and Allergy Research (https://www.meduniwien.ac.at/web/en/forschung/researcher-profiles/researcher-profiles/detail/?res=peter_pietschmann&cHash=8f4608341e5490f718d7844539788c5b).

### Collagen Sample Preparation

Collagen fibrils were extracted from the frozen mouse tails. The tails were first hydrated with PBS to thaw and then prepared according to our sample preparation procedure, which is sketched in detail in Figure S5.

First, the tail was carefully skinned and the tendons were extracted. Using a scalpel and precision tweezers, the tendons were further separated under a stereo microscope (Zeiss Stemi 508) to expose the collagen fibers. These fibers were then meticulously opened to reveal the collagen fibrils, which were extracted onto a 10 min plasma-cleaned (Plasma Cleaner PDC-002 (230 V) from Harrick) glass coverslip. After preparing the samples on the coverslips, the samples were rigorously washed with deionized water for at least 30 s to remove excess collagen material and salt residues. The prepared samples were then dried using nitrogen gas and stored in an airtight chamber until the experiment.

### Antibody Labeling

Prior to antibody labeling of the collagen fibrils, a custom-made fluid cell was mounted onto the coverslips by using two-component dental glue. The fluid cell was specifically designed with wells large enough to accommodate the AFM glass block holding the cantilever while featuring sufficiently high walls to prevent any liquid spillage during measurements, ensuring a controlled and stable environment for imaging. Once the fluid cell was in place, a small scratch was made on the underside of the coverslip, serving as a positional reference marker, enabling accurate alignment when the samples were transferred between the different setups.

Next, the collagen fibrils were labeled for 1 h at room temperature, using primary antibodies conjugated with Alexa Fluor 647. For labeling, we diluted anti-collagen1­(I) antibodies 1:1000 in PBS containing 1% BSA. After labeling, the staining solution was removed, and the samples were carefully rinsed with deionized water to clear the samples from excess dye. The samples were then fixed at room temperature for 15 min, using a 4% concentration of paraformaldehyde (PFA) diluted in deionized water. Following fixation, the PFA solution was removed, and the samples were thoroughly rinsed with deionized water to eliminate any residual PFA. Finally, PBS was carefully applied to the samples to maintain fibril hydration prior to imaging.

### Single Molecule Localization Microscopy

A home-built experimental setup was used, as described previously.^6a^ It is based on an Olympus IX73 (Japan) microscope body equipped with a high NA objective (Carl Zeiss, alpha-plan apochromat, 1.46 NA, 100×, Germany) and an EMCCD camera (Andor iXon Ultra). Furthermore, it features a red excitation laser (640 nm laser light, 100 mW nominal laser power, OBWAS Laser box, Coherent, USA). The setup was operated in spinning TIRF excitation (iLas^2^), yielding an excitation intensity of 1 kW/cm^2^. Lasers were filtered by a quad dichroic mirror (Di01-R405/488/532/635, Semrock, USA) and an emission filter (ZET405/488/532/642m, Chroma, USA) placed in the upper deck of the microscope body. Illumination and image acquisition were operated by VisiView (Visitron Systems, Germany). To maintain constant focus during imaging, a focus-hold system was built based on an IR laser, which was totally reflected at the surface of the coverslip. The reflected beam was then captured by an IR camera. Any vertical (*z*-axis) movement of the sample causes a lateral shift in the beam on the IR camera chip.^25^ A *z*-piezo underneath the objective was used both for focus-holding and for deliberate defocusing of the sample, as required for determining the *z*-positions of the single molecule signals (see subsection “Data Analysis for Single Molecule Localization Microscopy” below).

Fluorescent beads were added to the sample to serve as fiducial markers. The beads were sonicated in an ultrasonic bath for 2 min, diluted 1:100,000 in PBS, and finally applied to the sample for 5 min. Afterward the sample was rinsed with PBS. For dSTORM imaging,^26^ PBS was exchanged with blinking buffer consisting of 10% glucose, 500 μg/mL glucose oxidase, 40 μg/mL catalase, and 50 mM cysteamine in PBS (pH 7.5). The dSTORM buffer was always prepared fresh immediately prior to imaging or replaced between measurements, as its pH tends to shift after 1 h, potentially negatively affecting imaging quality.^27^

For dSTORM imaging, the first step was to select a suitable region of interest (ROI). Each ROI contained at least two collagen fibrils featuring an intersection but was free of excessive overlapping fibrils. While overlapping fibrils can theoretically be imaged using STORM, they usually have heights that exceed the TIRF excitation range and are more susceptible to movement, which renders them unsuitable for the imaging process. Any overlapping fibrils were therefore excluded from further analysis. After identification of an ROI, the *x* and *y* coordinates of the microscope stage were recorded, together with the reference marker, to enable precise relocation of the same ROI during subsequent AFM measurements. Next, the fluorophores within the selected ROI were excited in epifluorescence mode by using the red laser until the majority entered a dark state, demonstrating blinking behavior. For dSTORM imaging, the illumination time was set to 20 ms, the camera delay was adjusted to 21 ms, the excitation was switched to TIRF mode, and the camera gain was adjusted to 300.

The intensity of the red laser was maintained at 1 kW/cm^2^, while the intensity of the UV laser (wavelength of 405 nm) was set to 5% of the nominal power of 100 mW. The UV laser was used to excite the fluorescent beads used as fiducial markers every 100th recorded frame. Additionally, the UV laser aided in reactivating the fluorophores on the collagen, allowing for extended STORM recording durations.^1^ The sample was then defocused, typically 500 nm above the coverslip, unless specified otherwise. At least 80,000 images were recorded for each ROI; if the imaging time of a sample exceeded 50 min, the STORM buffer was exchanged for a fresh solution. Furthermore, we obtained the aberrations from the setup by measuring the psf from a *z*-stack of fluorescent beads with the same setup configuration that was used for the dSTORM measurements.

### Atomic Force Microscopy

After STORM imaging, the samples were washed, dried, and transferred to the AFM setup. The ROIs were relocated using the reference marker (glass scratch) and the recorded microscope stage positions. The samples were then imaged with the AFM, first under dry conditions in AC mode and afterward the samples were (re)­hydrated with PBS and imaged in Qi mode.

An AFM from JPK (Nanowizard 3, Bruker, Germany) was used with the following tips: (i) for measuring in AC mode: PPP-NCHR-50 (from Nanosensors), width: 30 μm, length: 125 μm, thickness: 4 μm, frequency: 330 kHz, spring constant: 42 N/m, tip radius: 7 nm; (ii) for recordings in liquid using the Qi-mode: MSCT-F (Bruker AFM Probes), width: 18 μm, length: 85 μm, thickness: 0.6 μm, frequency: 125 kHz, spring constant: 0.6 N/m, nominal tip radius: 10 nm, and typically a set point of 1.5 nN was chosen.

AFM images with a size of 20 μm × 20 μm and a pixel size of 39 × 39 nm were recorded to ensure sufficient information for comparison with the dSTORM data, which has a useable image size of approximately 30 μm × 30 μm. The camera connected to the AFM setup was a DMK 31BF03 Monochrome Camera, and the objective used for the AFM measurements was an air objective, Zeiss Plan-Neofluar 20×/0.5, 440340.

### Data Analysis for Single Molecule Localization Microscopy

The aberrations of the setup were calculated from the recorded bead data, using the Matlab application Aberration Measurement, which is available in the TU Wien Research Data repository (https://doi.org/10.48436/shmmj-7w030). For the localization of the fluorophores, the Python application mlefitgpu (https://github.com/jgmaloberti/mlefitgpu) was used. The software allows for prelocalization of the fluorophores, as well as modeling and fitting of psfs in order to determine the position of the fluorophores in 3D.

Fluorophores were prelocalized using their intensity values (absolute and relative pixel intensity). The psf was modeled using the imaging setup details and the calculated aberrations. The psf model allows for several input parameters, including the defocus, middle layer thickness, refractive index of the used media surrounding the fluorophores, and the refractive index of the middle layer (collagen).

To find the exact defocus value of a single ROI, a reference plane was first defined using only the fluorophores attached to the coverslip, adjacent to the collagen fibrils. Python codes for analysis are available under the following link: https://github.com/simonjaritz/SMLM-Analysis.git. The refractive index of the surrounding media was chosen to be *n*_w_ = 1.33 (water). The defocus is determined by setting the reference plane to *z* = 0 nm. Afterward, the height of the same fibril was determined from the AFM data, where the hydrated samples were imaged in Qi mode.

Using the determined defocus value and the reference height from the AFM, we reanalyzed the fibril using now an additional middle layer in our psf model. For the analysis of one fibril, we kept the height of the additional layer constant (fibril height from AFM), but varied its refractive index between 1.38 and 1.48. The individual fibrils were then further analyzed to obtain the cross sections and calculate their heights, which were then matched with the AFM data.

### Analysis of Collagen Cross Sections

The single molecule localizations were corrected for lateral drift using the recorded fiducial markers and tilt corrected using the reference plane at their determined defocus value. The localizations were further filtered, keeping only localizations with high fitting quality or low Log-Likelihood ratio (LLR < 600, with each localization featuring 15 × 15 pixels). The AFM images were first tilt corrected for each line. To find the contact point of the AFM tip on the collagen fibrils, force-distance curves of the Qi-measurements were fitted by a Hertz model by our in-house developed software (https://github.com/Rufman91/ForceMapAnalysis).

Both dSTORM and AFM measurements were further analyzed in order to show all localizations of a fibril in a single cross section. This analysis accounts for the curvature of the fibrils. First, the fibrils were fitted with a fourth-order spline (with *z* = 0) through their central axis, and then the shortest distance between the spline and each localization was calculated. All localizations were transformed to new coordinates denoted s, *X*, and *Z*, for the longitudinal, lateral, and vertical axes with respect to the fitted spline. The resulting cross sections feature all localizations along the fibril and were used for the fibrils’ height determination.

To calculate *H*_SMLM_ and *H*_AFM_, the localizations were cluster-filtered using the DBSCAN algorithm (available on https://scikit-learn.org/stable/modules/generated/sklearn.cluster.DBSCAN.html), to remove outliers. Next, the median height was calculated within a sliding window. The number of localizations per window was kept constant for each fibril, and the highest binned data point was taken as the height of the fibril. All steps of a single fibril analysis measured with SMLM are provided in Figure S6. For the determination of *H*_SMLM_, we further considered the precise location of fluorophores next to collagen fibrils, which defined the reference plane. The median height of debris at the coverslip surface was determined by AFM and added to *H*_SMLM_.

This process was repeated for each modeled refractive index, and the heights were then plotted against the refractive index together with the AFM height of a single fibril (see Figure S2c). Next, the intersection of a linear fit through the dSTORM data points and the AFM data was calculated, representing the refractive index of the fibril. Furthermore, the cross-sectional areas were calculated using the same sliding window binning method for AFM dry, *A*_dry_, and AFM wet, *A*_wet_, using numerical integration. Afterward, the refractive index was plotted against the swelling Δ*A*, defined as the relative cross-section area increase $\Delta A=\frac{A_{wet}}{A_{dry}}-1$ .

### Refractive Index Error Estimation

We used resampling to estimate the error of the height calculations, *H*_SMLM_ and *H*_AFM_. Briefly, half the localizations of a single cross-section were randomly selected, and the height was calculated for the selected data points. This process was repeated 1000 times, and the final standard deviation of the resulting Gaussian distribution was divided by $\sqrt{2}$ , yielding an error estimate for the collagen height.

For the error estimation of the refractive index, we employed random sampling from the obtained Gaussian distributions for the height estimates. This step was repeated 10,000 times to obtain the variance for the refractive index estimates σ_*n*,exp_^2^.

### Single Molecule Localization Precision

To calculate the localization precision, we tracked blinking signals of one and the same fluorophore over several frames, calculated the standard deviation of the determined positions, and used the standard error of the mean as the localization precision of the merged signals.

### Determination of the Refractive Index Measurement Precision

The statistical error of the refractive index measurement Δ*n* depends on the errors of the SMLM and AFM measurements. Because these errors are unrelated, the respective variances add up

2 $$\Delta n=\sqrt{\Delta n_{SMLM}^{2}\left(N,BG,\theta ,n,H\right)+\Delta n_{AFM}^{2}\left(\theta ,n,H,\Delta z_{AFM}\right)}$$

The error contribution of the SMLM measurement, Δ*n*_SMLM_, can be derived using CRLB analysis. While in SMLM, calculations of the CRLB are usually utilized to determine the localization precision; we follow an analogous analysis here to quantify the best precision to which the refractive index of the sample can be determined by any unbiased estimator, in case we know its true height *H*. For this we defined a function *I*_*x*,*y*_(*N*,BG,θ,*n*,*H*) that calculates images of single fluorescent molecules, i.e., numbers of photons detected in camera pixels with indices *x*,*y*. The function is based on the physical model published by Axelrod^16^ and computes a single molecule image by adding the intensity images of three orthogonally oriented emission dipoles. The function depends on the fluorescence signal *N* and the background level BG, several microscope-related parameters, such as the peak emission wavelength, numerical aperture, camera properties, etc., which are contained in a parameter vector θ; and finally, the refractive index *n* and height *H* of the sample. The function and a demonstration of its use can be found in the Python file “Single molecule image computation demo.py” under the following link: https://github.com/jgmaloberti/mlefitgpu.

The minimal error of the refractive index estimation caused by SMLM is then derived as the square root of the inverse Fisher information

3 $$\Delta n_{SMLM}=\sqrt{\frac{1}{FI}}$$

with the Fisher information calculated as

4 $$FI=\sum_{x,y}\frac{1}{I_{x,y}}{\left(\frac{\partial I_{x,y}}{\partial n}\right)}^{2}$$

where we do not show the explicit dependencies of FI and *I*_*x*,*y*_ on the many arguments for clarity.

To determine the influence of the AFM measurement error Δ*z*_AFM_ on the error of the refractive index measurement, Δ*n*_AFM_, we calculated (noise-free) molecule images *I*_*x*,*y*_ assuming sample thicknesses *H* ± Δ*z*_AFM_, and identified those refractive index values that provide the best-matching (noise-free) images under the assumption that the sample thickness is *H*.More precisely, we performed the following optimization

5 $${\mathrm{n̂}}_{\mp }=\arg\min_{n_{fit}}\left({\left|I_{xy}\left(n,H\pm \Delta z_{AFM}\right)-I_{xy}\left(n_{fit},H\right)\right|}^{2}\right)$$

From this, we approximated a symmetric error Δ*n*_AFM_ as

6 $$\Delta n_{AFM}=\pm \frac{1}{2}\left(\left|n-{\mathrm{n̂}}_{-}\right|+\left|n-{\mathrm{n̂}}_{+}\right|\right)$$

which was feasible since $\left|n-{\mathrm{n̂}}_{-}\right|$ and $\left|n-{\mathrm{n̂}}_{+}\right|$ showed only negligible differences.

## Supplementary Material



## Data Availability

A preprint version of this paper is available at bioRxiv: Jaritz, Simon, Lukas Velas, Anna Gaugutz, Manuel Rufin, Philipp J. Thurner, Orestis G. Andriotis, Julian G. Maloberti, Simon Moser, Alexander Jesacher, and Gerhard J. Schütz. “Refractive index mapping below the diffraction limit via single molecule localization microscopy”. bioRxiv (2025): 2025-08. https://www.biorxiv.org/content/10.1101/2025.08.20.670782v1, accessed November 21, 2025. All localization data, analysis scripts, and a detailed description of the software used are provided in the TU Wien Research Data repository (https://doi.org/10.48436/shmmj-7w030).

## Notes

Weak dependence of Δ*n*·*H* on the collagen thickness, an example of a collagen fibril cross section imaged with SMLM and AFM, comparison between the ground truth height measured via AFM and the apparent height determined via SMLM as a function of the assumed refractive index, application of refractive index mapping to a second fibril, calculation of the required numbers of signal photons *N*_min_ as a function of sample thickness *H*, and sketch showing the collagen fibril sample preparation (PDF)
