# Supplementary material for: Refractive Index Mapping below the Diffraction Limit via Single Molecule Localization Microscopy
Source: ACS Nano. 2025 Dec 26;20(1):1335–44. doi: 10.1021/acsnano.5c17647 (PMC12822553; doi:10.1021/acsnano.5c17647)
Supplement: Supplementary file 1 [file nn5c17647_si_001.pdf]

Supporting Information

Refractive index mapping below the diffraction limit via single molecule localization microscopy

Authors: Simon Jaritz[1], Lukas Velas[1], Anna Gaugutz[1], Manuel Rufin[2], Philipp J. Thurner[2], Orestis G. Andriotis[2], Julian G. Maloberti[3], Simon Moser[3], Alexander Jesacher[3], Gerhard J. Schütz[1, ‡]

(1) Institute of Applied Physics, TU Wien, 1060 Vienna, Austria
(2) Institute of Lightweight Design and Structural Biomechanics, TU Wien, 1060 Vienna, Austria
(3) Institute of Biomedical Physics, Medical University of Innsbruck, Müllerstraße 44, 6020 Innsbruck, Austria
‡ Please send correspondence to: schuetz@iap.tuwien.ac.at

Supporting Figures

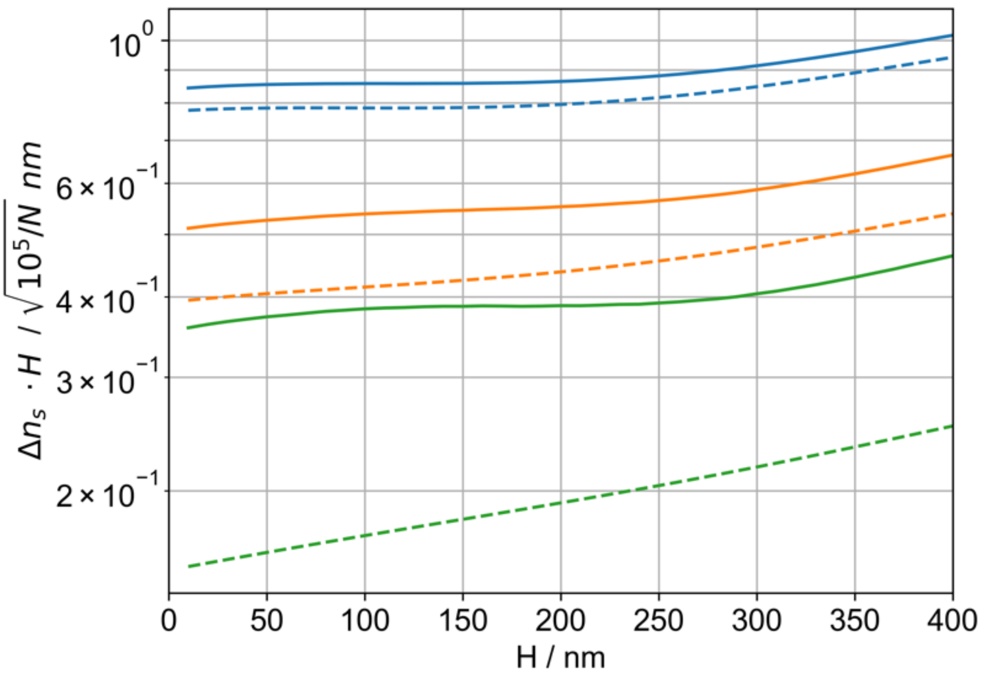

Figure S1: The product of $\Delta n \cdot H$ depends only weakly on the collagen thickness. The plot was calculated for the same parameter settings as **Fig.1d**, i.e. a refractive index of $n = 1.42$ and a signal of 10^5 photons. Color code is identical to **Fig.1d**.

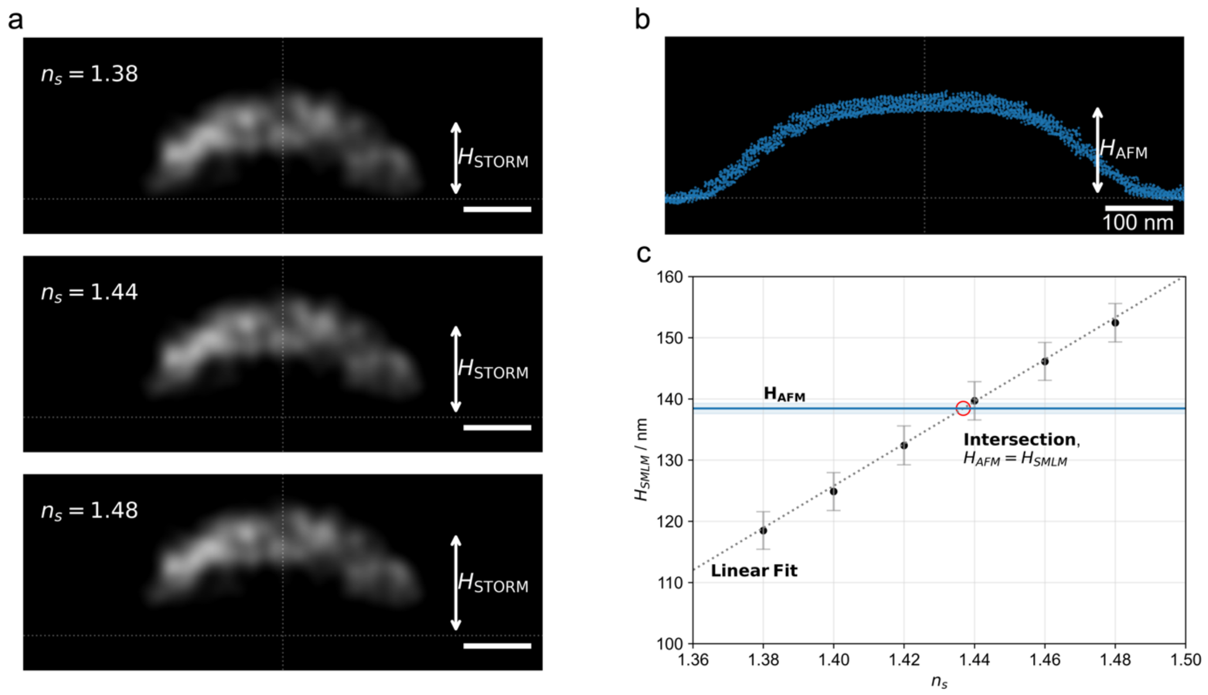

Figure S2: Example of a collagen fibril cross section, imaged with **a** SMLM and **b** AFM in hydrated state. SMLM data were analysed with different assumptions about the refractive index, shown here exemplarily for $n_s = 1.38, 1.44, 1.48$. The AFM profile yielded $H_{AFM} = 138$ nm. Scale bar = 100 nm. **c** Comparison between the ground truth height measured *via* AFM (H_{AFM},) and the apparent height determined *via* SMLM (H_{SMLM}) as a function of the assumed refractive index (n). From the intersection, the correct refractive index of the collagen fibril can be determined. Shown are data obtained from a single collagen fibril. For this fibril we calculated a refractive index $n_{collagen} = 1.437 \pm 0.003$.

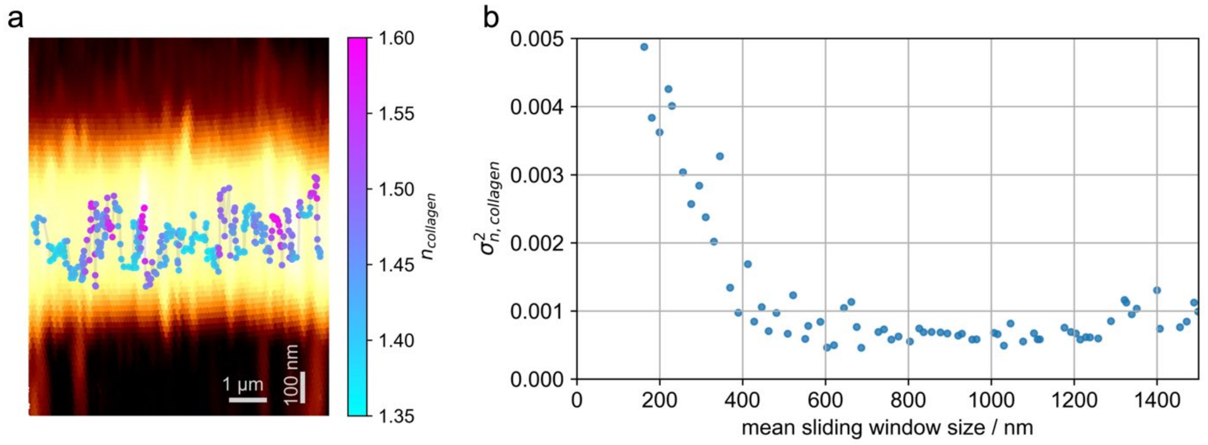

Figure S3: a Overlay of the AFM image from an example fibril and the refractive index $n_{collagen}$ along the central fibril axis. Each datapoint was calculated from a cross-sectional profile within a sliding window containing 100 localisations, with a 90% overlap between windows; the mean sliding window size was 200 nm. In each window, H_{SMLM} was determined as described in the Methods section. Each dot was plotted at the calculated mean position of all localizations within each window. **b** Variance of the refractive index along the fibril shown in panel **a** for different sizes of the sliding window. Experimentally determined variances, σ_n^2, were corrected for the expected errors in determining the refractive index, $\sigma_{n,exp}^2$, according to

$\sigma^2_{n,collagen} = \sigma^2_n - \sigma^2_{n,exp}$. $\sigma^2_{n,exp}$ was calculated separately for each sliding window (see Methods section).

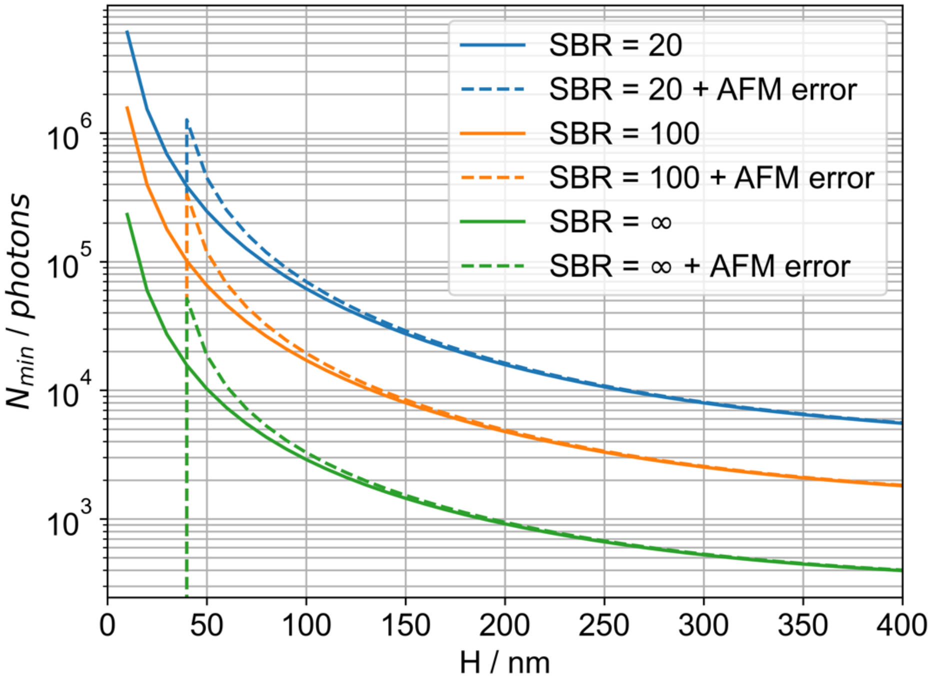

Figure S4: Required numbers of signal photons N_{min} as a function of sample thickness H, to ensure a refractive index precision of $\Delta n_s = 10^{-2}$ assuming an ideal AFM (solid lines) and an AFM error of 1 nm (dashed lines).

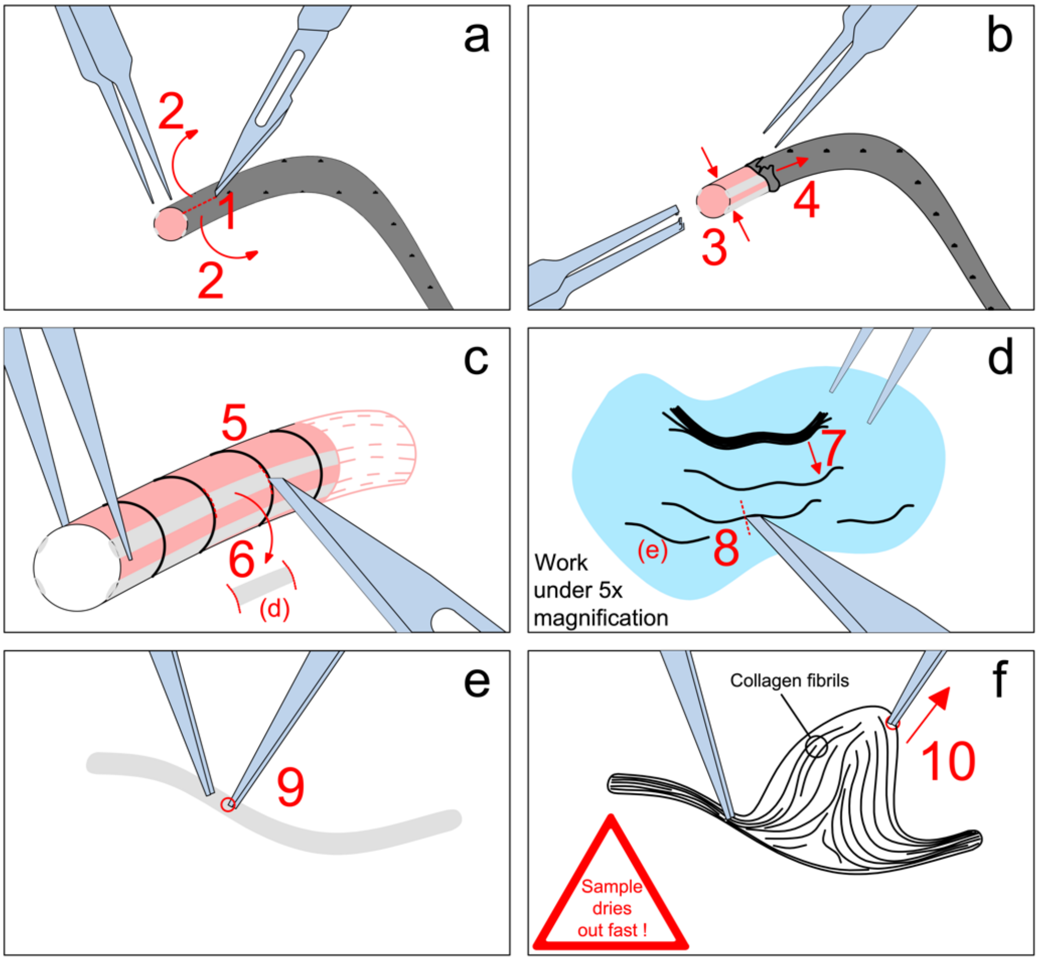

Figure S5: Collagen fibril sample preparation. **a** Cut lengthwise (approximately 1 cm) with a scalpel (1) and pull the skin towards the end of the tail using tweezers (2). **b** Hold the proximal end of the tail tightly with the toothed, thumbed forceps (3) and pull the skin in the distal direction using either tweezers or an additional pair of toothed, thumbed forceps (4). **c** Make perpendicular cuts in the tendons, approximately 1 cm apart (5). Be careful not to cut the muscle tissue underneath and to the sides of the tendons. Next, remove the cut tendon pieces with tweezers and a scalpel (6). Those tendon pieces can then be stored in the freezer till they are used for fibril sample preparation (step **d**) and onwards. Alternatively, a clamp with three teeth can be used to fixate the tail in place and the tendon can be cut out. The cutting is performed utilizing a scalpel and a pair of tweezers similarly as described before. **d** Place a piece of tendon into a round petri dish (Ø 3.5 cm) and hydrate with PBS or sterile water. Place the sample under the stereo microscope using 5X magnification. Use a pair of tweezers to separate the fibres (7) and the scalpel to cut them (8), to get a higher sample yield. Each separated fibre can then be used for the next step (9). **e** Take a piece of fibre and place it onto a 10-minute plasma cleaned coverslip. Next, pinch it with the tip of precision tweezers (9). **f** Pull gently on the rest of the fibril with a second pair of precision tweezers to reveal the collagen fibrils (10). This step has to be performed fast (within a few seconds) as the fibres are prone to drying out.

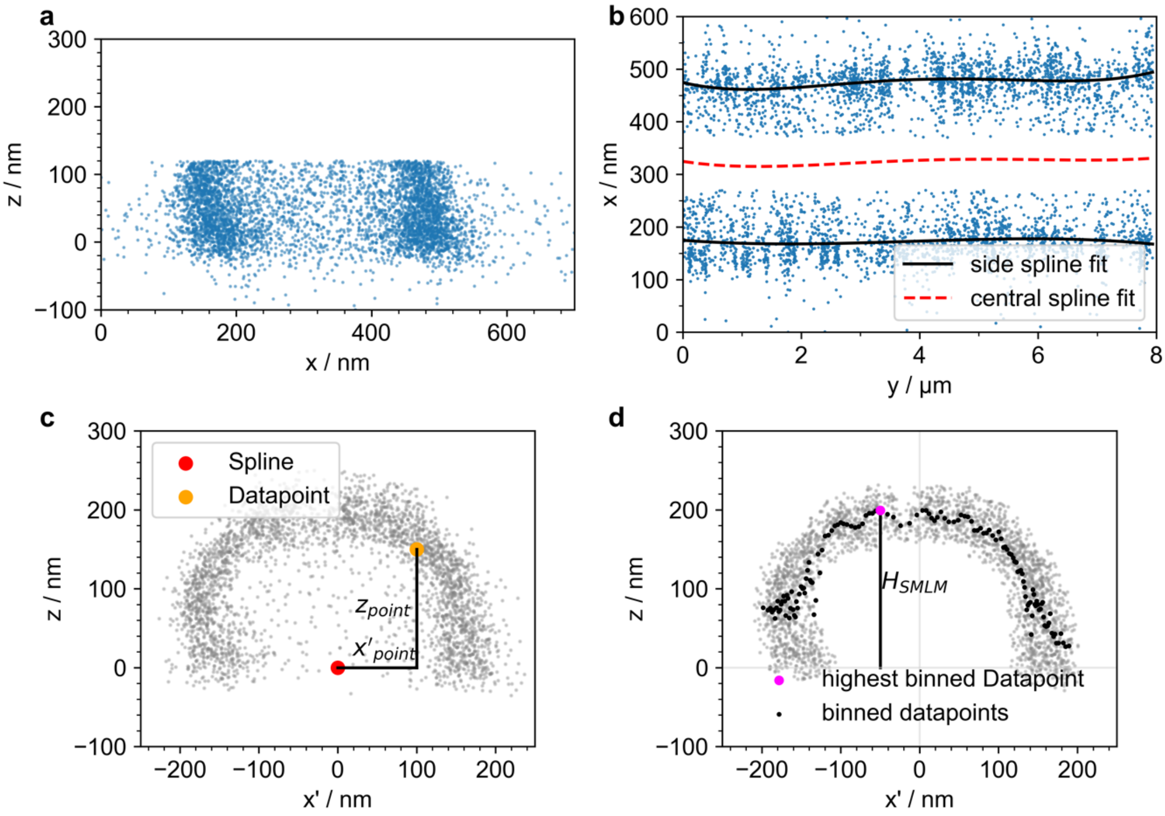

Figure S6: Procedure for height determination of collagen fibrils imaged with SMLM. We first rotated the fibril such that its long axis aligned roughly with the y-axis of the coordinate system. The z-axis was chosen in the vertical direction. **a** Cross-sectional profile of a single fibril projected onto the (x, z) plane. Data were cropped at half maximum to highlight the fibril rims. **b** Top view of the same fibril. We selected the two rims of the fibril and fitted the data with 4th order 2-dimensional splines. Next, a central spline was fitted through the two rim splines, which was taken as the collagen as the collagen axis for subsequent analysis. This procedure ensured that localization clusters due to overcounting of single molecule signals had little impact on the fitted central spline. **c** Transformed cross-sectional profile of the fibril. Datapoint coordinates were transformed, with x' denoting the horizontal distance from the central spline. **d** To determine the fibril height from the transformed cross-section, we removed outliers using DBSCAN and binned the datapoints along the x'-axis using a sliding window. In this example, each window contained 40 localizations and featured an overlap of 50%. Binned data points are shown in black. For determination of the height, H_{SMLM}, we used the highest binned datapoint (magenta).
